# Ubiquitin C-terminal hydrolase-L1 increases cancer cell invasion by modulating hydrogen peroxide generated via NADPH oxidase 4

**DOI:** 10.18632/oncotarget.3843

**Published:** 2015-04-15

**Authors:** Hyun Jung Kim, Venkataraman Magesh, Jae-Jin Lee, Sun Kim, Ulla G. Knaus, Kong-Joo Lee

**Affiliations:** ^1^ Graduate School of Pharmaceutical Sciences, College of Pharmacy, Ewha Womans University, Seoul, Korea; ^2^ Conway Institute, University College Dublin, Dublin, Ireland

**Keywords:** UCH-L1, ubiquitination, hydrogen peroxide, NOX4, invasion

## Abstract

This study explored the role of ubiquitin C-terminal hydrolase-L1 (UCH-L1) in the production of ROS and tumor invasion. UCH-L1 was found to increase cellular ROS levels and promote cell invasion. Silencing UCH-L1, as well as inhibition of H_2_O_2_ generation by catalase or by DPI, a NOX inhibitor, suppressed the migration potential of B16F10 cells, indicating that UCH-L1 promotes cell migration by up-regulating H_2_O_2_ generation. Silencing NOX4, which generates H_2_O_2_, with siRNA eliminated the effect of UCH-L1 on cell migration. On the other hand, NOX4 overexpressed in HeLa cells happens to be ubiquitinated, and NOX4 following deubiquitination by UCH-L1, restored H_2_O_2_-generating activity. These *in vitro* findings are consistent with the results obtained *in vivo* with catalase (−/−) C57BL/6J mice. When H_2_O_2_ and UCH-L1 levels were independently varied in these animals, the former by infecting with H_2_O_2_-scavenging adenovirus-catalase, and the latter by overexpressing or silencing UCH-L1, pulmonary metastasis of B16F10 cells overexpressing UCH-L1 increased significantly in catalase (−/−) mice. In contrast, invasion did not increase when UCH-L1 was silenced in the B16F10 cells. These findings indicate that H_2_O_2_ levels regulated by UCH-L1 are necessary for cell invasion to occur and demonstrate that UCH-L1 promotes cell invasion by up-regulating H_2_O_2_ via deubiquitination of NOX4.

## INTRODUCTION

Metastasis is the phenomenon in which tumor cells break away from a primary site, circulate through the bloodstream, infiltrate vascular and lymphatic vessels, and settle and proliferate elsewhere in the body [1-3]. This process involves the interplay of malignant cell- and host-associated factors [4], including the microenvironment of the extravasated cancer cells and factors that promote the survival and growth [5, 6] of the tumor cells. These factors are believed to include ROS [7-10] such as hydrogen peroxide (H_2_O_2_), superoxide (O_2_^·-^), and hydroxyl radical (OH·), which are up-regulated in the tumor microenvironment. Various studies support the role of ROS in cancer, angiogenesis [11], mitogenesis, and resistance to apoptosis [12, 13]. Introduction of oncogenes into immortalized cells leads to increases in cellular ROS, and ROS-generating genes such as NADPH oxidases (NOX). NOX have been shown to contribute to tumorigenesis and angiogenesis [14-17]. Of special interest, inhibition of NOX4 suppressed the growth of melanoma cells, indicating that NOX4-generated ROS are required for transformation of melanoma cells [18].

Levels of ubiquitin-C-terminal hydrolase-L1 (UCH-L1), which catalyzes hydrolysis of C-terminal ubiquitin esters and amides, increase in various cancers [19-23], especially during tumor invasion and metastasis [24, 25]. This raises the question, whether the roles played by ROS and UCH-L1 in tumor-cell invasion and metastasis are interrelated. In this study, we explored this question *in vitro* in murine metastastic melanoma (B16F10) and HeLa cells, and also *in vivo* in a catalase (−/−) mouse model. We confirmed that H_2_O_2_ regulates tumor invasion and that UCH-L1 significantly increases both cell migration and H_2_O_2_ generation. Both processes were attenuated when H_2_O_2_ was removed using Adv-catalase, or by treatment with the NOX inhibitor DPI, or by inhibiting ROS generation using NOX4 siRNA. Also, we demonstrated that UCH-L1 restores H_2_O_2_-gernerating activity of NOX4 by deubiquitinating NOX4. These findings suggest that UCH-L1 plays a key role in tumor invasion by modulating the H_2_O_2_ generating NOX4 activity.

## RESULTS

### UCH-L1 affects cellular ROS generation

In a previous study, we showed that UCH-L1 plays a key role in lung metastasis [25], but we did not explore the underlying mechanism. Since ROS play important roles in tumor progression, and in pro-metastatic signaling pathway [8, 26], we investigated whether UCH-L1 is involved in ROS-mediated cell invasion.

**Figure 1:**
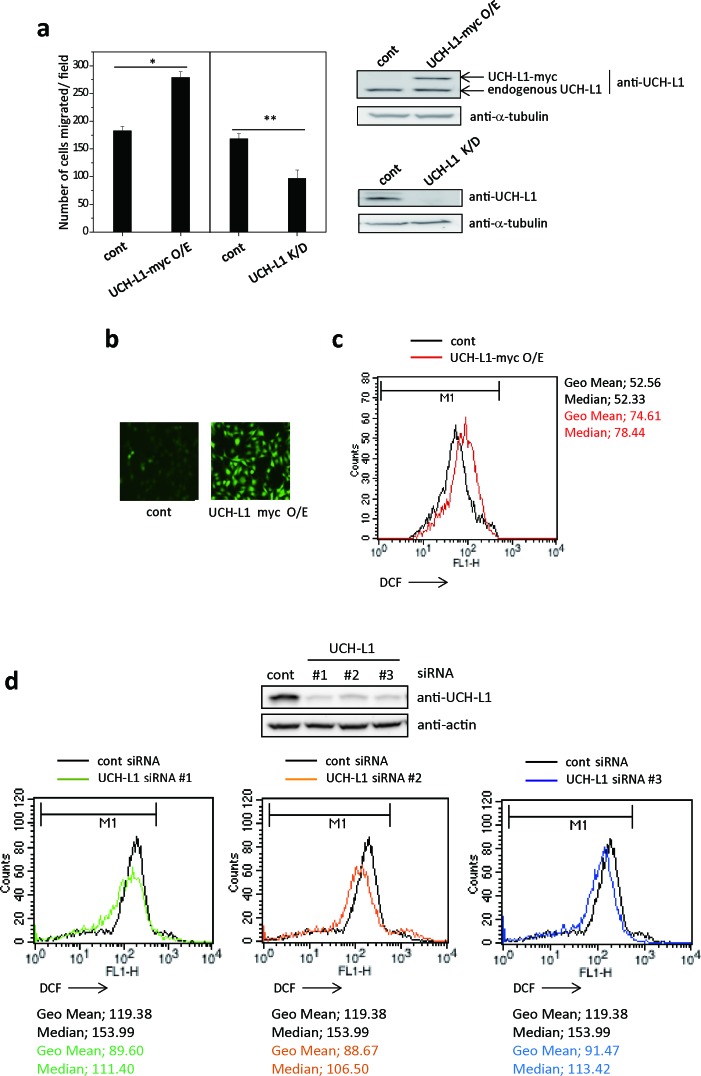
UCH-L1 enhances cellular ROS generation **a.** B16F10 cells expressing UCH-L1 (UCH-L1-myc O/E), UCH-L1 shRNA (UCH-L1 K/D) or control vector (cont) were immunoblotted for UCH-L1, using tubulin as control. Cells (1 × 10^4^) were seeded on matrigel-coated inserts in transwell chamber and incubated at 37ÐC for 24 h. Cells migrated through the filter were counted. Data are mean ± SD (*n* = 3). *P < 0.05 for cont *vs*. UCH-L1 O/E, **P < 0.05 for cont vs. UCH-L1 K/D. **b.** Cellular ROS levels were determined by fluorescence microscopy using CM-H_2_DCFDA. **c.** For flow cytometry, equal numbers of cells were treated with 3 μM CM-H_2_DCFDA in HBSS at 37°C for 15 min and immediately, the fluorescence intensity was measured. **d.** B16F10 cells were transfected with UCH-L1 specific siRNAs (#1-3) for 48h. Equal numbers of cells were treated with 3 μM CM-H_2_DCFDA in HBSS at 37°C for 15 min and then the fluorescence intensity was measured by flow cytometry. Geometric mean (Geo Mean) fluorescence intensity and Median value of histogram are calculated by statistical analysis of BD CellQuest software.

First, we generated stable UCH-L1-overexpressing- or UCH-L1-knocked down-B16F10 cells and compared their invasiveness using transwell chambers coated with matrigel *in vitro*. As shown in Figure 1a, B16F10 cells overexpressing UCH-L1 showed increased ability for invasion, while knocking-down UCH-L1 decreased their invasiveness. These results suggest that there is a positive correlation between UCH-L1 expression levels and cell invasion. Next, we examined whether UCH-L1 expression is associated with cellular ROS generation, assessing cellular ROS levels by measuring fluorescence generated by ROS after loading the cells with CM-H_2_DCFDA. B16F10 cells overexpressing UCH-L1 showed increased cellular ROS levels (Figure 1b and 1c), while knocking it down with various siRNA decreased the cellular ROS levels (Figure 1d). These results indicate that UCH-L1 increases cellular ROS generation.

### UCH-L1 is involved in H_2_O_2_-mediated cell invasion

Recent studies reported that H_2_O_2_ might cooperate with TGF-β to induce the metastatic phenotype of HCC cells [27]. Induction of the metastatic phenotype is accompanied by increases in steady-state H_2_O_2_ that drives pro-migratory signaling [28].

**Figure 2:**
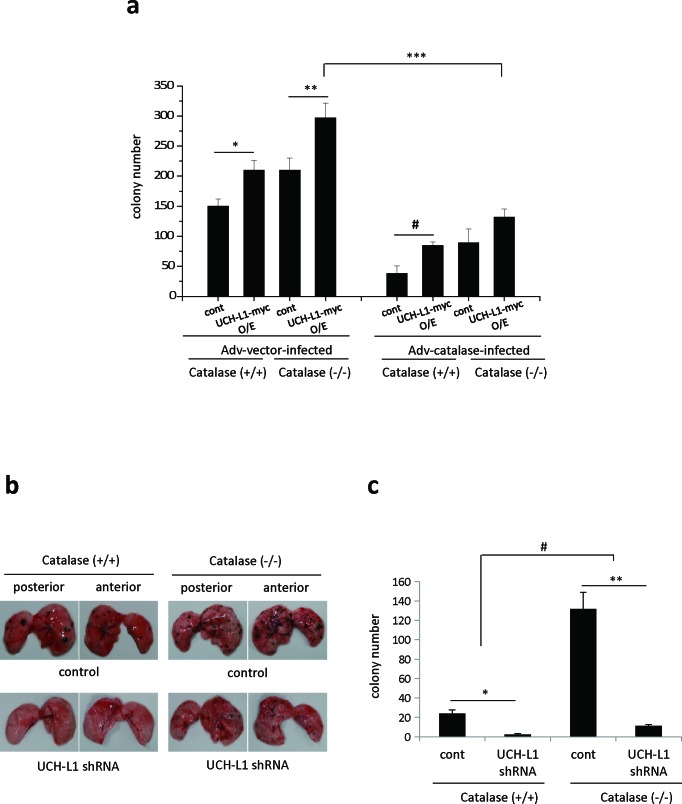
UCH-L1 is involved in H_2_O_2_-mediated pulmonary metastasis *in vivo* **a.** UCH-L1 overexpressing (UCH-L1-myc O/E) or control (cont) B16F10 cells were infected with Adv-vector (Adv-vector-infected) and Adv-catalase (Adv-catalase-infected) (1 × 10^5^ plaque forming units (PFU)). Cells (1.0 × 10^6^) were i.v. injected into tail vein of male C57BL/6 catalase (+/+) mice and catalase (−/−) mice. Two weeks after i.v. injection, the lung was extirpated, and the black spherical B16F10 colonies were counted. Data are mean ± SD (*n* = 7). *P < 0.05 for catalase (+/+) (Adv-vector-infected cont) *vs*. catalase (+/+) (Adv-vector-infected UCH-L1 O/E), **P < 0.05 for catalase (−/−) (Adv-vector-infected cont) *vs*. Catalase (−/−) (Adv-vector-infected UCH-L1 O/E), ^#^*P* < 0.05 for catalase (+/+) (Adv-catalase-infected cont) *vs*. catalase (+/+) (Adv-catalase-infected UCH-L1 O/E), ***P < 0.05 for catalase (−/−) (Adv-vector-infected UCH-L1 O/E) *vs*. catalase (−/−) (Adv-catalase-infected UCH-L1 O/E). **b.** *In vivo* pulmonary metastasis assay by injecting B16F10 cells (1.0 × 10^6^) knocked down UCH-L1 (UCH-L1 shRNA) or control cells intravenously into the tail vein of male C57BL/6 catalase (+/+) mice and catalase (−/−) mice. The images were photographed immediately without fixation after being extirpated. **c**. The results of pulmonary metastasis were presented in bar graph. Two weeks after i.v. injection, the lung was extirpated, and the black spherical B16F10 colonies were counted. Data are mean ± SD (*n* = 5-7). *P < 0.05 for catalase (+/+) (cont) *vs*. catalase (+/+) (UCH-L1 shRNA), **P < 0.05 for catalase (−/−) (cont) *vs*. catalase (−/−) (UCH-L1 shRNA), ^#^*P* < 0.05 for catalase (+/+) mice *vs*. catalase (−/−) mice.

We examined whether UCH-L1 is involved in H_2_O_2_-mediated cell invasion *in vivo*, by assessing pulmonary metastasis in catalase (−/−) mice, and in B16F10 cells stably overexpressing UCH-L1, infected with Adv-catalase. Because catalase is a specific H_2_O_2_ scavenging enzyme, catalase (−/−) mice exhibit lower ability to consume extracellular H_2_O_2_ in lung and liver [29]. As shown in Figure 2a, pulmonary metastasis of B16F10 cells overexpressing UCH-L1 is increased in both catalase (−/−) and catalase (+/+) mice relative to that of control cells. This effect is greater in catalase (−/−) mice than in catalase (+/+) mice. Pulmonary metastasis of B16F10 cells overexpressing UCH-L1 was attenuated in mice injected with Adv-catalase-infected B16F10 cells by up to 75%, compared to that in mice injected with Ad-vector-infected B16F10 cells. Additionally, pulmonary metastasis of UCH-L1-knocked-down B16F10 cells was significantly less than that of control cells in both catalase (−/−) and catalase (+/+) mice (Figure 2b and 2c). These results indicate that the effect of knocking down UCH-L1 on pulmonary metastasis of B16F10 cells is similar to that of infection with Adv-catalase, and that UCH-L1 is involved in H_2_O_2_–mediated cell invasion by altering H_2_O_2_ levels in invasive cells. We also found that metastasis of B16F10 cells to the lungs of catalase (−/−) mice (right upper panel in Figure 2b) significantly increased compared to that in catalase (+/+) mice (left upper panel in Figure 2b). This finding indicates that metastasis is higher in catalase (−/−) mice, and that the H_2_O_2_ levels in the host microenvironment are also important for the metastatic process.

### Adv-catalase infection attenuates the enhancing effect of UCH-L1 on cell invasion *in vitro*

**Figure 3:**
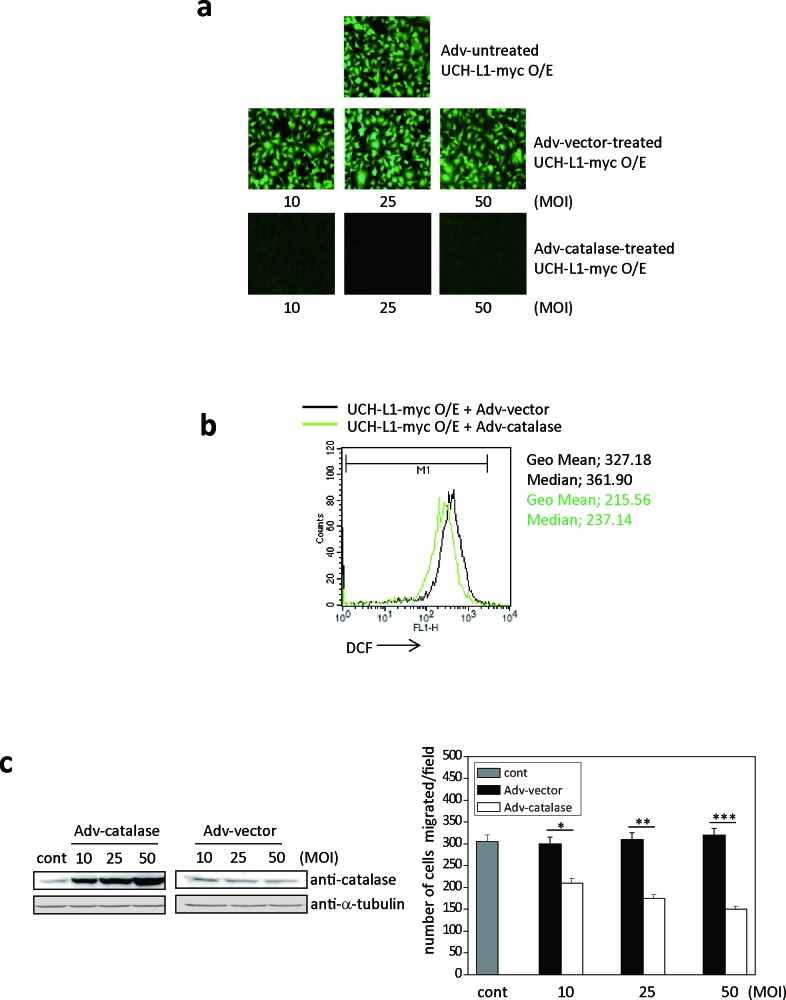
Adv-catalase attenuates the positive effect of UCH-L1 on cell invasion *in vitro* **a**. Cellular ROS levels of UCH-L1-overexpressing B16F10 cells infected with Adv-vector (10, 25, 50 MOI) or Adv-catalase (10, 25, 50 MOI) were determined by fluorescence microscopy using CM-H_2_DCFDA. **b**. UCH-L1-overexpressing B16F10 cells were treated with Adv-vector or Adv-catalase (25 MOI) and incubated with 3 μM of CM-H_2_DCFDA in HBSS at 37°C for 15 min and analyzed by flow cytometry. Geometric mean (Geo Mean) fluorescence intensity and Median value of histogram are calculated by statistical analysis of BD CellQuest software. **c**. After infection with Adv-vector or Adv-catalase, B16F10 cells were immunoblotted for catalase with tubulin as control, and seeded on transwell chambers to determine migration. Data are mean ± SD (*n* = 3). *P < 0.05 for Adv-vector *vs*. Adv-catalase (10 MOI), **P < 0.05 for Adv-vector *vs*. Adv-catalase (25 MOI), ***P < 0.05 for Adv-vector *vs*. Adv-catalase (50 MOI).

To confirm that UCH-L1 promotes pulmonary metastasis *in vivo* by altering H_2_O_2_ levels in the invasive cells, we examined whether UCH-L1-induced ROS generation is blocked by Adv-catalase infection in a MOI-dependent manner (10-50 MOI) *in vitro*. As shown in Figure 3a, H_2_O_2_ in B16F10 cells overexpressing UCH-L1 was significantly attenuated when cells were infected with Adv-catalase, but not Adv-vector. The fluorescence intensity (geometric mean (Geo Mean)) of cells infected with Adv-catalase, assessed using FACS, was less than that of cells infected with Adv-vector (Figure 3b).

Moreover, the migration of B16F10 cells overexpressing UCH-L1 was significantly reduced after infection with Adv-catalase in a MOI-dependent manner (Figure 3c). These results demonstrate that UCH-L1 overexpression leads to enhanced H_2_O_2_ generation, and that invasion induced by UCH-L1 is reduced by eliminating H_2_O_2_, suggesting that UCH-L1 plays a role in the regulation of H_2_O_2_ generation.

### NOX4 siRNA attenuates UCH-L1-mediated H_2_O_2_ generation and cell invasion

It has been reported that NOX4 promotes proliferation and metastasis of non-small lung cancer cells [30] and induces epithelial-to-mesenchymal transition and migration of breast epithelial cells [31]. Additionally, the ROS released from NOX4 was shown to be H_2_O_2_ [32-35].

To determine whether the likely source of ROS in B16F10 cells overexpressing UCH-L1 is NOX family of NADPH oxidases, we first examined the effect of DPI, a flavoprotein inhibitor of NOX, on ROS generation in these cells. After exposing the cell lines to 20 μM of DPI for 30 min, their cellular ROS levels and migratory capacities were measured *in vitro*. We found that both the generation of ROS (Supplementary Figure 1A) and migration (Supplementary Figure 1B) of B16F10 cells overexpressing UCH-L1 were significantly attenuated by DPI. This suggests that UCH-L1 mediates ROS generation via NOXs in B16F10 cells overexpressing UCH-L1.

**Figure 4:**
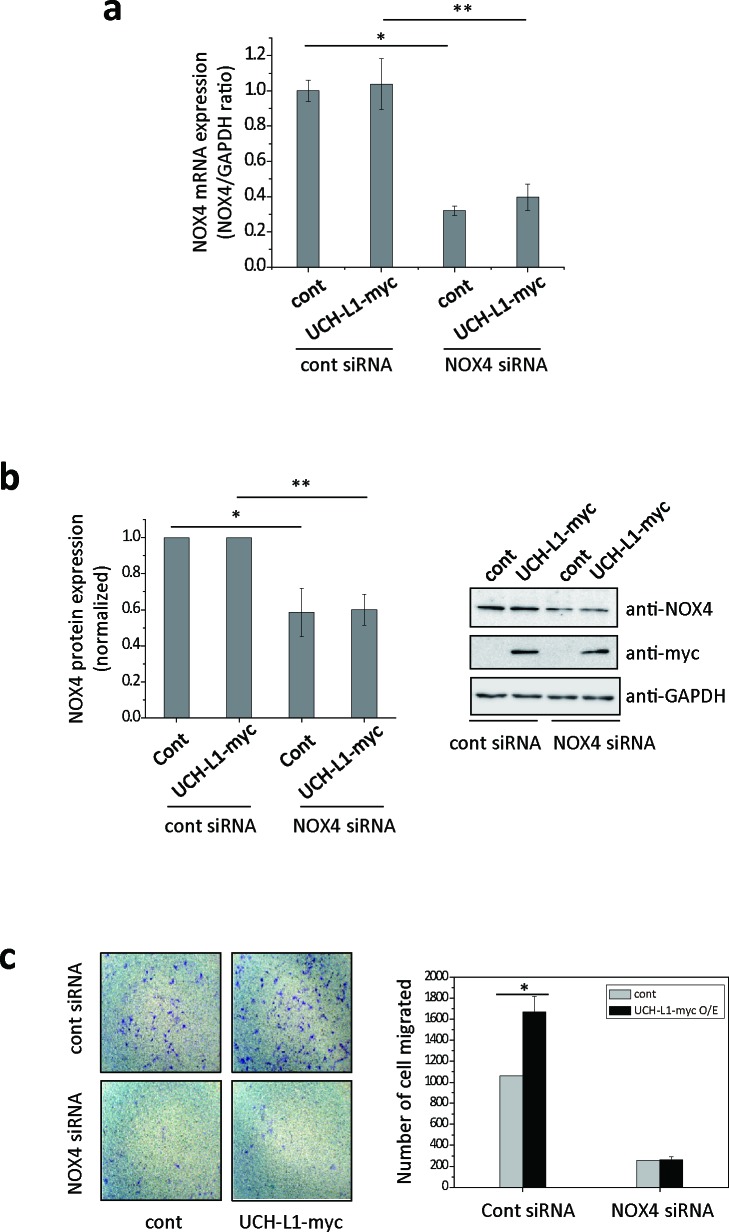
NOX4 siRNA attenuates UCH-L1-mediated H_2_O_2_ generation and cell invasion **a**. B16F10 cells were transfected with NOX4 siRNA, followed by analysis of NOX4 mRNA expression by quantitative real-time RT-PCR. The quantity of NOX4 mRNA was normalized to the quantity of a house keeping gene, *GAPDH*. Data are mean ± SD (*n* = 3). *P < 0.05 for cont (cont siRNA) *vs*. cont (NOX4 siRNA), **P < 0.05 for UCH-L1 O/E (cont siRNA) *vs*. UCH-L1 O/E (NOX4 siRNA). **b.** NOX4 protein expression was determined using western blotting with anti-NOX4 antibody. Data are mean ± SD (*n* = 3). *P < 0.05 for cont (cont siRNA) *vs*. cont (NOX4 siRNA), **P < 0.05 for UCH-L1 O/E (cont siRNA) *vs*. UCH-L1 O/E (NOX4 siRNA). **c**. B16F10 cells were transfected with NOX4 siRNA, seeded on transwell chambers and incubated at 37ÐC for 24 h. The invading cells were counted. Data are mean ± SD (*n* = 3). *P < 0.05 for cont (cont siRNA) *vs*. UCH-L1 O/E (cont siRNA).

Next, to test the possibility that UCH-L1 can modulate NOX4 causing increased H_2_O_2_ generation, we investigated whether UCH-L1 regulates NOX4-dependent cell migration using NOX4 specific siRNA. As shown in Figure 4a and 4b, NOX4-specific siRNA significantly decreased both NOX4 mRNA and protein levels, while overexpression of UCH-L1 did not affect either. Knocking down NOX4 in B16F10 cells significantly reduced both basal and UCH-L1 enhanced invasiveness of cells (Figure 4c), suggesting that both inherent and UCH-L1-enhanced invasiveness of B16F10 cells depend on NOX4.

### UCH-L1 decreases ubiquitinated NOX4

Because UCH-L1 hydrolyses ubiquitin C-terminal, and because NOX4 contains Lys (K) residues in the structural regions implicated in NOX4 catalytic activity [34, 36], we wondered whether NOX4 activity is regulated by post-translational modifications such as ubiquitination or deubiquitination.

First, we examined whether UCH-L1 regulates the expression of NOX4 mRNA or NOX4 protein [32]. As shown in Supplementary Figure 2A, no differences were found in NOX4 mRNA expression between UCH-L1-overexpressing and UCH-L1 knocked down B16F10 cells. The amount of NOX4 protein expressed in B16F10 cells overexpressing UCH-L1 was also not significantly different from that in B16F10 cells in which UCH-L1 was knocked down (Supplementary Figure 2B). Additionally, we examined whether UCH-L1 regulates NOX4 degradation by treating the cells with cycloheximide, an inhibitor of protein synthesis. No difference was observed in endogenous NOX4 in UCH-L1-overexpressing and control cells (Supplementary Figure 2C). These results suggest that UCH-L1 does not influence the expression of NOX4.

**Figure 5:**
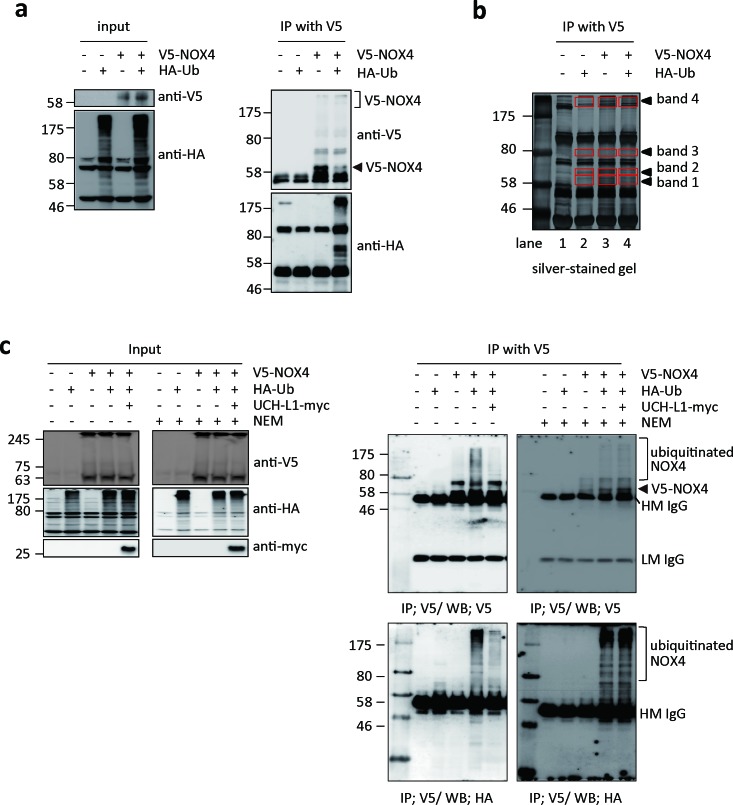
UCH-L1 deubiquitinates NOX4 **a**. HeLa cells were transfected with V5-NOX4 and/or HA-tagged ubiquitin (HA-Ub) and control expression vectors. Levels of ubiquitination of NOX4 were determined by western blotting of immunoprecipitates with anti-HA antibody, while NOX4 expression was monitored with anti-V5 antibody. **b**. For mass spectrometry analysis, immunoprecipitates were separated on 10% SDS-PAGE gel, silver-stained, and then analyzed by nanoUPLC-ESI-Q-TOF MS as described in “Methods”. Red boxes indicate the analyzed bands in each lane as shown in Table 1. **c**. HeLa cells were transfected with V5-NOX4, HA-Ub or UCH-L1-myc expression vectors as indicated. Cells were lysed in IP buffer without or with 10 mM of NEM to inhibit UCH-L1 and then immunoprecipitated with anti-V5 antibody. Levels of ubiquitinated NOX4 and total protein levels of V5-NOX4, HA-Ub, and UCH-L1-myc were determined by western blotting.

Next, we examined whether NOX4 can be ubiquitinated and deubiquitinated by UCH-L1 in HeLa cells, which lack endogenous UCH-L1 [37], and in which NOX4 is barely detectable by western blotting with anti-NOX4 antibody. HeLa cells transfected with V5-NOX4 expression vector with or without HA-Ub expression vector were immunoprecipitated with V5 antibody and the precipitate was analyzed by western blotting using anti-V5 and anti-HA antibodies. As shown in Figure 5a, ubiquitinated NOX4 was detected only in cells co-transfected with both V5-NOX4 and HA-Ub expression vectors. To further confirm that NOX4 is ubiquitinated (shown in Figure 5a right lower panel), NOX4 was immunoprecipitated using anti-V5 antibody from bigger batch of samples in Figure 5a, the immune complexes separated on SDS-PAGE and detected with silver-staining (Figure 5b). Newly appearing protein bands 1-4 (red boxes) were identified by peptide sequencing using nanoUPLC-ESI-q-TOF tandem MS after digestion of the proteins in the bands with trypsin. Lane 2 corresponding to only HA-Ub served as control for V5-NOX expression in lanes 3 and 4. Proteins and peptides identified in this experiment are listed in Table 1, after excluding the non-specific proteins identified in control lane 2 from the list of proteins in lanes 3 and 4. As expected, NOX4, ubiquitin, and Lys-48 linkage ubiquitin chain were verified at bands 3 and 4 in the lane 4.

**Table 1:** Proteins identified in immune complex of NOX4 in Figure 5b by nanoUPLC-ESI-q-TOF tandem mass spectrometry

lane	transfection	Band no.	Protein	Accession no.	Observed ms (CS)	Caculated ms	Delta M	Mascot score	peptide matched	Modification
2	HA-Ub	Band 1	N.D.							
		Band 2	N.D.							
		Band 3	N.D.							
		Band 4	N.D.							
3	V5-NOX4	Band 1	Nox 4	Q7Z7G3	1128/0526(2+)	2254.1845	−0.0938	24	DLLLPPSSQDSEIUPFIQSR	
					474.7578(2+)	947.5328	−0.0317	18	LLFDELAK	
		Band 2	N.D.							
		Band 3	Ubiquitin	P62988	534.3415(2+)	1066.6135	0.055	51	ESTLHLVLR	
		Band 4	ND.							
4	HA-Ub + VS-NOX4	Band 1	Nox 4	Q7Z7G3	1128/0204(2+)	2254.1845	4.1582	26	DLLLPPSSQDSEIUPFIQSR	
					474.7406(2+)	947.5328	40661	15	LLFDELAK	
		Band 2	N.D.							
		Band 3	Nox 4	Q7Z7G3	488.2494(2+)	9749821	0.0021	19	IVGDWTER	
					1128.058(2+)	2254.1845	−0.083	22	DLLLPPSSQDSEIUPFIQSR	
			Ubiquitin	P62988	487.5997(3+)	1459.7783	−0.001	48	UFAGKQLEDGR	GlyGly (K48)
					541.2819(2+)	1080.5451	0.0041	37	TLSDYNIQK	
					534.3121(2+)	1066.6135	−0.0038	12	ESTLHLVLR	
		Band 4	Nox 4	Q7Z7G3	474.7249(2+)	947.5328	4.0975	42	LLFDELAK	
					705.3078(2+)	1408.735	47.134	32	LLFDELAKYNR	formyl (K495)
			Ubiquitin	P62988	487.5531(3+)	1459.7783	−0.1408	22	UFAGKQLEDGR	GlyGly (K48)
					534.2647(2+)	1066.6135	−0.0986	54	ESTLHLVLR	

N.D: not detected; CS: chargestate; Observed ms: observed mass; Caculated on: calculated mass; Delta M; delta mass GlyGly (w): ubiquitination at lysine of identified peptide

We investigated whether ubiquitinated NOX4 is a target of UCH-L1, a deubiquitinating enzyme. Figure 5c shows that ubiquitination of NOX4 was markedly decreased in HeLa cells when UCH-L1-myc expression vector was co-transfected with V5-NOX4 and HA-Ub expression vectors. We further investigated whether endogenous NOX4 is also deubiquitinated by UCH-L1 in B16F10 cells. As shown in Supplementary Figure 3, UCH-L1 siRNA increases the level of ubiquitination of endogenous NOX4 in B16F10 cells. However, when the cells were treated with N-ethylmaleimide (NEM), an irreversible inhibitor of UCH-L1, ubiquitination of NOX4 increased (Figure 5c right lower panel). This was confirmed with UCH-L1 inactive mutant, C90S (Supplementary Figure 4). Immunoprecipitation studies confirmed that UCH-L1 deubiquitinates NOX4, suggesting that UCH-L1 might be involved in H_2_O_2_–mediated cell invasion by regulating the NOX4 activity through deubiquitination.

### UCH-L1 restores H_2_O_2_-generating activity of NOX4 by deubiquitinating NOX4

**Figure 6:**
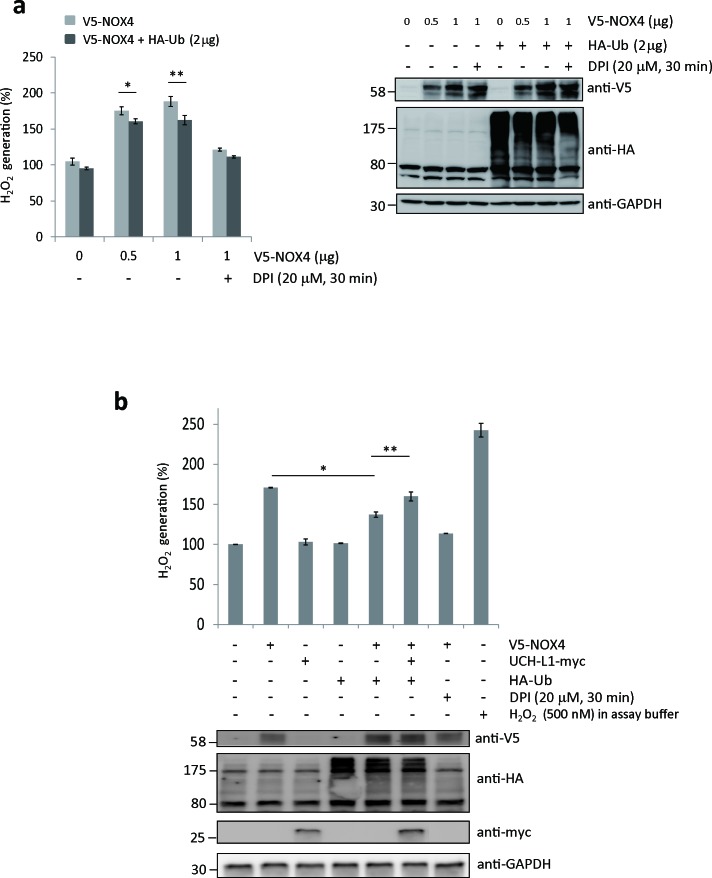
UCH-L1 restores H_2_O_2_-generating activity of NOX4 by deubiquitinating NOX4 **A**. HeLa cells were transfected with V5-NOX4 expression vector (0, 0.5, and 1.0 μg) in a dose-dependent manner with or without 2 μg of HA-Ub expression vector. H_2_O_2_ release was measured using Amplex^®^ Red. Cells treated with DPI (20 μM, 30 min) were used as a negative control. Levels of ubiquitinated NOX4 and total protein levels of HA-Ub and GAPDH were determined by western blotting. Data are mean ± SD (*n* = 3). *P < 0.05 for V5-NOX4 (0.5 μg) *vs*. V5-NOX4 (0.5 μg) + HA-Ub, **P < 0.05 for V5-NOX4 (1 μg) *vs*. V5-NOX4 (1 μg) + HA-Ub. **B**. H_2_O_2_ release was measured using Amplex^®^Red. Total protein levels of V5-NOX4, HA-Ub, UCH-L1-myc, and GAPDH were determined by western blotting. Cells treated with 20 μM of DPI for 30 min were used as a negative control. 500 nM of H_2_O_2_ in assay buffer was used as a positive control. Data are mean ± SD (*n* = 3). *P < 0.05 for cells transfected only with V5-NOX4 *vs*. cells transfected with both V5-NOX4 and HA-Ub, **P < 0.05 for cells transfected with both V5-NOX4 and HA-Ub *vs*. cells transfected with V5-NOX4, HA-Ub, and UCH-L1-myc.

To determine whether ubiquitination of NOX4 affects its H_2_O_2_-generating activity, we measured H_2_O_2_ generated from HeLa cells co-transfected with both V5-NOX4 and HA-Ub expression vectors using an Amplex Red-based fluorometric assay. As shown in Figure 6a, H_2_O_2_ release increased in a dose-dependent manner by expressing V5-NOX4, but significantly decreased when cells were co-transfected with V5-NOX4 and HA-Ub, indicating that H_2_O_2_-generating activity of NOX4 is in part negatively modulated by ubiquitination. In this experiment, cells treated with DPI were used as a negative control.

As shown in Figure 6b, the H_2_O_2_-generating activity of NOX4 decreased in cells transfected with both V5-NOX4 and HA-Ub, compared to cells transfected with V5-NOX4 alone. However, NOX4 activity was significantly restored when these cells were co-transfected with UCH-L1-myc. Cells treated with DPI and H_2_O_2_ in the assay buffer were used respectively as negative and positive controls. Because endogenous NOX4 protein levels in HeLa cells are not high enough for detection by western blotting, we could not detect the positive effect of UCH-L1 on H_2_O_2_-generating activity of NOX4 when HeLa cells were transfected with UCH-L1-myc vector alone (Figure 6b). These results suggest that UCH-L1 restores H_2_O_2_-generating activity of NOX4 through deubiquitination.

**Figure 7:**
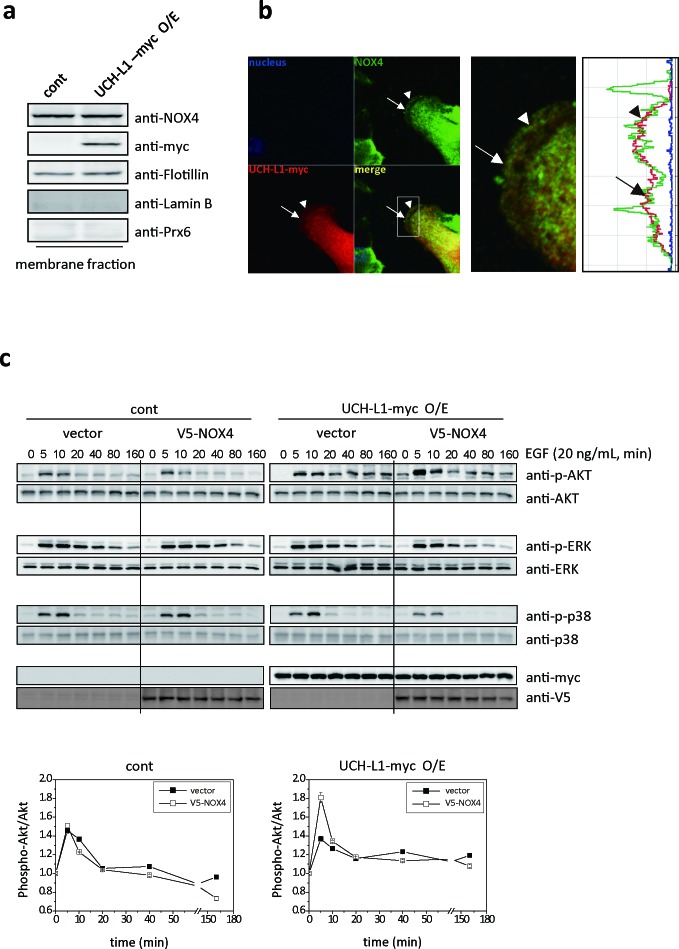
UCH-L1 co-localizes with NOX4 at leading edge of plasma membrane; and H_2_O_2_ induced by UCH-L1 via NOX4, causes Akt activation **A**. Immunoblotting of B16F10 cells after cell fractionation using anti-NOX4 and myc (for UCH-L1) antibodies. Anti-flotillin, anti-laminB, and anti-Prx6 antibodies respectively were used as fractionation markers; flotillin for membrane fraction, laminB for nucleus fraction, and Prx6 for cytosolic fraction. **B**. Localization of UCH-L1 and NOX4 in B16F10 cells was evaluated by fluorescence confocal microscope. White box was magnified to right panel with fluorescence intensity profiles. Arrow and arrow head indicate area of UCH-L1 and NOX4 co-localization in the merge image. **C**. HeLa cells overexpressing UCH-L1 were transiently transfected with V5-NOX4 expression vector and then starved for 6 h. Cells were then treated with EGF (20 ng/mL) for various times (0, 5, 10, 20, 40, 80, and 160 min). At the indicated time points, EGF-treated cells were lysed and analyzed by western blotting using phospho-ERK (P-ERK), phospho-p38 (P-p38), and phospho-Akt (P-Akt) antibodies. Quantitative analysis was done with multi-gauge software (LAS 3000). Data are mean ± SD (*n* = 3).

We also found that UCH-L1 is colocalized close enough to endogenous NOX4 in B16F10 cells to be deubiquitinated. Co-localization of UCH-L1 and NOX4 was found at the leading edge of plasma membrane in B16F10 cells, using western blot analysis (Figure 7a), and immunocytochemical-staining (Figure 7b). To confirm the cross contamination of membrane fractions in Figure 7a, anti-Flotillin, anti-Lamin B, and anti-Prx6 antibodies were used as reagents for detection of membrane, nucleus, and cytosolic fraction, respectively.

### H_2_O_2_ induced in UCH-L1 via NOX4, activates Akt through EGF-induced signal transduction

To understand how UCH-L1-mediated H_2_O_2_ regulates cell invasion, we examined the kinetics of activation of various kinases including Akt and MAPKs. The Akt and MAPK family can be activated downstream of growth factor receptor kinases [25, 38, 39]. HeLa cells overexpressing UCH-L1 were transiently transfected with empty vector or V5-NOX4 vector, and exposed to EGF (20 ng/mL) for various durations. The observed activation kinetics of Akt, ERK, and p38 are shown in Figure 7c. Activation of Akt in HeLa cells overexpressing UCH-L1 was significantly higher in cells transiently transfected with V5-NOX4, compared to that in cells transfected with vector. No differences were detected in activation of ERK and p38. These results confirm that the activation of Akt is regulated by UCH-L1- induced H_2_O_2_ through NOX4 in HeLa cells.

## DISCUSSION

In the present study, we have shown that UCH-L1 deubiquitinates NOX4, thereby up-regulating its ability to generate H_2_O_2_ and promoting the invasive potential of B16F10 cells both *in vitro* and *in vivo.* We further show that pulmonary metastasis is down-regulated in catalase (−/−) mice injected with B16F10 cells in which UCH-L1 is knocked down with specific shRNA (Figure 2b and 2c). We conclude that UCH-L1 is involved in the NOX4-mediated H_2_O_2_ generation. A previous study [40] on the role of UCH-L1 in tumor metastasis focused only on UCH-L1 expression levels in various tumors including neuroblastoma, pancreatic cancer, but not the levels of H_2_O_2_ induced by UCH-L1. This study is therefore the first to demonstrate the involvement of UCH-L1 in H_2_O_2_-mediated tumor metastasis.

ROS such as H_2_O_2_, superoxide (O_2_·^−^) and hydroxyl radical (OH·), have been shown to be up-regulated in the tumor microenvironment and trigger cell adhesion, cell migration and invasion [41]. NOX4 has been detected in focal adhesions [42], and on the plasma membrane [43]. Recently, it has been reported that NOX4 promotes non-small cell lung cancer cell proliferation and metastasis through positive feedback regulation of PI3K/Akt signaling [30] and is involved in TGF-beta and SMAD3-driven induction of the epithelial-to-mesenchymal transition and migration of breast epithelial cells [31]. It has also been reported that NOX4-generated ROS are required for promoting hypoxia-induced invasive potential of U87 cells, and that ROS are potential targets for inhibiting tumor cell invasion and infiltration in glioblastoma [44]. A recent report demonstrated that invasiveness of lung carcinoma H460 cells is regulated by ROS [41], and that OH· promotes the regulation of cancer cell migration. While this report identified OH· as a positive modulator of lung carcinoma H460 cell migration, the present study shows that UCH-L1 induces H_2_O_2_ generation by modulating NOX4 activity and that H_2_O_2_ plays an important role in the invasion of B16F10 cells.

A recent study demonstrated that NOX4 is negatively regulated by hydrogen peroxide inducible clone-5 (Hic-5) protein [45], and also that NOX4 protein expression is suppressed by Hic-5 via Cbl-c and HSP27-mediated ubiquitination and proteasomal degradation. Our quantitative real time RT-PCR analysis revealed that UCH-L1 does not affect the levels of NOX4 mRNA or protein expression. Also, we showed that ubiquitination down-regulated the catalytic activity of NOX4, while deubiquitination by UCH-L1 increased this activity. These findings point to UCH-L1 as yet another regulator of NOX4 activity. Further studies are required to understand the NOX4 deubiquitination mechanism by UCH-L1, because little is known of UCH-L1 action mechanism except for the demonstrated hydrolase action on ubiquitin-C-terminal adducts amides and esters [46].

Signal transduction conducted by the microenvironment around the primary tumor may trigger tumor metastasis, especially at the migration stage. Sustained mitogen activated protein kinase (MAPK)- signaling involved in uncontrolled tumor cell migration, depends on crosstalks among integrin, receptor tyrosine kinase (RTK) and protein kinase C (PKC) [39]. H_2_O_2_ has been shown to contribute to the MnSOD-promoted invasion in glioma cells through activation of Akt and ERK [47]. Here we have shown that UCH-L1-mediated H_2_O_2_ generation via NOX4, influences the activation of Akt in HeLa cells overexpressing UCH-L1, indicating that such H_2_O_2_ generation increases migration of B16F10 cells by modulating Akt. This is consistent with our previous finding in H157 cells that UCH-L1 increases cell migration by modulating Akt activation [25].

In studies on pulmonary metastasis of B16F10 cells in catalase (−/−) mice, we found evidence that H_2_O_2_ plays an important role in metastasis, that UCH-L1-induces H_2_O_2_ and that increased cellular H_2_O_2_ levels are critical for up-regulation of pulmonary metastasis. This study is also the first to establish H_2_O_2_ as an essential factor for this metastatic process.

**Figure 8:**
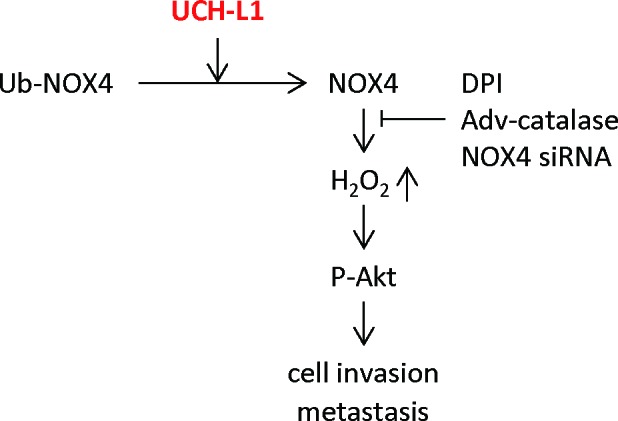
Suggested pathway linking UCH-L1-mediated H_2_O_2_ generation to increased cell invasion

In summary, we demonstrated that UCH-L1 promotes H_2_O_2_ generation by up-regulating NOX4 activity through deubiquitination, and that H_2_O_2_, so produced, plays an important role in cell invasion *in vitro* by regulating the upstream kinase Akt (Figure 8). Employing the catalase (−/−) mouse model, we also demonstrated that UCH-L1-induced H_2_O_2_ acts as an essential metastatic factor *in vivo*.

## MATERIALS AND METHODS

### Cell culture and transfection

B16F10 melanoma and HeLa cells were grown at 37°C in DMEM and EMEM supplemented with 100 units/mL of penicillin G, 100 μg/mL streptomycin and 10% fetal bovine serum (all from Invitrogen: CA, USA), respectively. Use of human NOX4 expression plasmid, pcDNA3.0-V5-NOX4, was described in a previous report [36]. UCH-L1 was cloned into pcDNA3.1-myc/his(−)A vector (Invitrogen, CA, USA) to generate pcDNA3.1-UCH-L1-myc. Transient transfections were performed using Effectene^®^ transfection reagent (Qiagen, CA, USA) according to the manufacturer's instructions.

### Immunoprecipitation and western blot analysis

Cells (2 × 10^6^) were lysed in an IP buffer (50 mM Tris-HCl, 150 mM NaCl, 1 mM EDTA (pH 7.4), 0.5% sodium deoxycholate, 1% Triton X-100, 0.1% SDS) containing protease inhibitor cocktail (Sigma-Aldrich, USA) and phosphatase inhibitors (5 mM Na_3_VO_4_) for 10 min on ice, sonicated for 5 seconds twice, and centrifuged at 12,000 rpm for 10 min. The supernatant was incubated with anti-V5 antibody for 2 h at 4°C and then with protein G Sepharose^TM^ affinity beads (GE Healthcare Bioscience AB, Uppsala, Sweden) for 1 h at 4°C. The beads were washed six times each with 1 mL of the IP buffer to remove nonspecific binding. The immune complex was solubilized in SDS gel sample buffer, separated by 10% SDS-PAGE, and detected with silver staining (Silver staining kit: GE Healthcare Bioscience AB, Uppsala, Sweden) or western blot analysis. Chemiluminescence signal was captured using LAS3000 system (Fujifilm, Japan) and each band was quantified using Multi Gauge V3.0 software (Fujifilm, Japan). The sources of antibodies used in this study were: anti-UCH-L1, and anti-myc antibodies - Millipore (MA, USA); anti-HA antibody - Roche (USA); anti-phospho-Akt (P-Akt), anti-phospho-p38 (P-p38), anti-phospho-ERK (P-ERK) antibodies - Cell Signaling Technology (MA, USA); anti-V5 antibody - Invitrogen (CA, USA); anti-α-tubulin antibody - Santa Cruz Biotechnology (CA, USA). Anti-NOX4 antibody was provided from YS Bae (Ewha Womans University, Korea) [48] or purchased from Abcam (Cambridge, USA) and Proteintech Group (IL, USA).

### Generation of B16F10 stable cell lines

B16F10 cells stably overexpressing UCH-L1 were generated using LentiM1.4 lentiviral vector (Macrogen: Seoul, Korea). Cells were infected with lentiviral transduction particles carrying UCH-L1 sequence with C-terminal myc tag (Accession No. NM_004181) or with control lentiviral vector in complete culture medium with polybrene (8 μg/mL) for 5 h. The cells were cultured for 7 days in puromycin-containing medium for selection. B16F10 cells with stable knockdown of UCH-L1 were generated using shLenti1.1 lentiviral vector (Macrogen: Seoul, Korea). Lentiviral transduction particles carrying shRNA sequence against UCH-L1 [49] or control non-target sequence (AATCGCATAGCGTATGCCGTT) were used to knock down UCH-L1 expression. For selection, Zeocin^TM^-containing culture medium was used for 14 days.

### Adenoviral-catalase infection

B16F10 cells were infected with adenovirus encoding human catalase (Adv-catalase) or empty adenoviral vector (Adv-vector) (AbClon: Seoul, Korea) in DMEM containing 2% FBS, overnight at 37°C. Catalase expression was monitored with anti-catalase antibody (AbClon: Seoul, Korea).

### Down-regulation of UCH-L1 using UCH-L1 specific siRNA

UCH-L1 specific siRNAs were obtained from RNAi libraries predesigned by BIONEER Co. (Daejeon, Korea). Cat No. 1443992 for UCH-L1 siRNA #1 (BIONEER, Korea); Cat No. 1443993 for UCH-L1 siRNA #2 (BIONEER, Korea); Cat No. 1443994 for UCH-L1 siRNA #3 (BIONEER, Korea); sense 5′-TTCTCCGAACGTGTCACGT-3′ and antisense 5′-ACGTGACACGTTCGGAGAA-3′ for control siRNA. Transfections with UCH-L1 siRNA #1, 2 and 3 were carried out using Lipofectamine^®^ RNAiMAX (Invitrogen: CA, USA) according to the manufacturer's protocol.

### Down-regulation of NOX4 mRNA and protein expression using NOX4 specific siRNA

NOX4 specific siRNAs were obtained from YS Bae (Ewha Womans University, Korea) [50] and RNAi libraries predesigned by BIONEER Co. (Daejeon, Korea). These were designed as follows: sense 5′-GTAGGAGACTGGACAGAAC-3′ and antisense 5′-GTTCTGTCCAGTCTCCTAC-3′ for NOX4 siRNA #1; Cat No. 1392780 for NOX4 siRNA #2 (BIONEER, Korea); Cat No. 1392781 for NOX4 siRNA #3 (BIONEER, Korea); sense 5′-TTCTCCGAACGTGTCACGT-3′ and antisense 5′-ACGTGACACGTTCGGAGAA-3′ for control siRNA. We ascertained the decreased levels of H_2_O_2_ in B16F10 cells transfected with NOX4 siRNA #1, 2, or 3, using Amplex^®^ Red hydrogen peroxide assay kit (data not shown). We used NOX4 siRNA #1 for our study.

### Quantitative studies on real-time reverse transcription-polymerase chain reaction

Total RNAs were extracted from cells with RNeasy^®^ Mini Kit (Qiagen: Hilden, Germany) and reverse-transcribed by SuperScript^TM^ II reverse transcriptase with Oligo dT primers (Invitrogen: CA, USA). The resultant cDNAs for mouse NOX4 were quantitatively amplified with real-time RT-PCR using QuantiTect^®^ Primer Assay kit (Cat No; QT00126042 for NOX4, Qiagen, Hilden, Germany) and DyNAmo^TM^ HS SBYR^®^green qPCR kit (Thermo scientific: MA, USA). Standard curve was obtained by plotting Ct (cycle threshold) values against log cDNA concentrations of five serial dilutions of the target nucleic acid. GAPDH was used as an internal control.

### Measurement of cellular ROS

Cellular ROS levels were measured using the fluorescent dye, 2′, 7′-dichlorodihydrofluorescein diacetate (CM-H_2_DCFDA) (Molecular Probes: OR, USA). Cells were incubated with 5 μM CM-H_2_DCFDA in Hank's balanced salt solution (HBSS) at 37°C for 5 min. The plate was mounted, and the DCF fluorescence images were immediately acquired by fluorescence microscopy, Axiovert 200 (Carl Zeiss: Jena, Germany). For flow cytometry, equal numbers of cells were harvested, washed with cold PBS, and treated with 3 μM CM-H_2_DCFDA in HBSS at 37°C for 15 min and the cellular ROS was immediately measured using FACSCalibur flow cytometer (BD Biosciences: NJ, USA). To quantify the log-amplified fluorescence which is emitted by cells, geometric mean (Geo Mean) of the fluorescence intensity as well as the Median value for the fluorescent peak was calculated by statistical analysis of BD CellQuest software. For the experiment using DPI, equal numbers of cells were treated with 20 μM of DPI for 30 min.

### Hydrogen peroxide (H_2_O_2_)-generating activity assay

H_2_O_2_-generating activity was measured in intact cells using Amplex^®^ Red hydrogen peroxide assay kit (Molecular Probes, OR, USA) [51]. Transfected cells (2~3 × 10^5^) were added to 200 μL of assay buffer (25 mM HEPES (pH 7.4) containing 0.12 M NaCl, 3 mM KCl, 1 mM MgCl_2_, 0.1 mM Amplex Red, and 0.032 unit HRP) and incubated for 60 min at 37°C. Fluorescence intensity was measured using Molecular Devices Spectramax Gemini EM Fluorescence Microplate Reader (excitation wavelength: 544 nm; emission wavelength: 590 nm). For a positive control, 500 nM of H_2_O_2_ in assay buffer was used. For a negative control, cells treated with 20 μM DPI for 30 min were used.

### Cell invasion assay

This was performed using a 24-well Transwell^®^ unit with polycarbonate membrane (pore size, 8 μm) (Corning: NY, USA). The membrane was coated with Matrigel^TM^ basement membrane matrix (1 μg/μL) (BD Bioscience, NJ, USA). Cells (0.5~1 × 10^4^) were seeded into the upper chamber in a serum-free medium. The lower chamber was filled with a medium containing 10% FBS. After incubation for 24 h at 37°C, the cells on the upper side of membrane were removed with a cotton swab. The cells invading to the underside of the membrane were stained with 0.5% w/v Crystal violet in 25% methanol and counted at 100-fold magnification under a microscope (Carl Zeiss, Jena, Germany).

### Protein identification using nanoUPLC-ESI-q-TOF tandem MS

To identify the proteins and modifications, the gels were destained and digested in gel with trypsin, and extracted as previously described [52]. The resulting peptides were dissolved in 10% acetonitrile containing 0.1% formic acid and subjected to nanoAcquity™ UPLC™/ESI/q-TOF tandem MS (SYNAPT™ HDMS™, Waters Co. UK), desalted on line using trap column (i.d. 180 μm × 20 mm, Symmetry^®^ C18) cartridge, and separated on a C18 reversed-phase 75 μm i.d. × 200 mm analytical column (1.7 nm particle size, BEH130 C18, Waters Co. UK) with integrated electrospray ionization PicoTip^TM^ (10 μm i.d., New Objective, USA) using nanoAcquity™ UPLC/ESI/q-TOF MS/MS. Ten μL of peptide solutions in buffer A (water/formic acid; 100 : 0.1, v/v), were injected onto a column and eluted by a linear gradient of 5-60% buffer B (ACN/formic acid; 100:0.1, v/v) over 100 min. Initially, the flow rate was set to 250 nL/min and a capillary voltage of 2.8 keV was applied to the UPLC™ mobile phase before spray. Chromatography was performed on line to SYNAPT™ HDMS™. The mass spectrometer was programmed to record scan cycles composed of one MS scan followed by MSMS scans of the 3 most abundant ions in each MS scan. MS parameters for efficient data-dependent acquisition were: intensity (>10), number of components to be switched from MS to MS/MS analysis. Tandem MS (MS/MS) spectra were matched against amino acid sequences in SwissProt human database (version 57.8., 20401 entries) using Mascot search (version 2.2.06) and MOD^i^ [53]. The search parameters were: 0.3 Da tolerance for peptide and fragment ions; digestion with trypsin with up to one missed cleavage allowed. Acetylation (N-terminal), formylation (Lys), deamidation (Asn and Gln), oxidation (Met), phosphorylation (Ser, Thr, and Tyr), pyro-Glu modification (N-terminal Glu and N-terminal Gln), and ubiquitination (Lys) were the searched variable modifications.

### Confocal microscopy

UCH-L1 overexpressing and control B16F10 cells were seeded on SecureSlip™ Silicone Supported coverglass (Grace bio-labs: OR, USA). After fixing with 4% paraformaldehyde in HBSS for 10 min, cells were permeabilized by incubation with 0.1% Triton X-100 in HBSS for 10 min. To block non-specific protein adsorption, cells were treated with HBSS buffer containing 3% BSA, 0.2% Tween 20, and 0.2% gelatine. Next, we used mouse monoclonal anti-myc antibody and AlexaFluor 568 conjugated goat anti mouse IgG (Molecular Probes: OR, USA) for UCH-L1-myc, and rabbit polyclonal anti-NOX4 antibody and AlexaFluor 488 conjugated goat anti-rabbit IgG (Molecular Probes: OR, USA) for NOX4. DAPI was used for nuclear staining. Images were taken at 40 × magnification with a fluorescence confocal microscope, LSM510 (Carl Zeiss, Jena, Germany).

### Catalase (–/–) and catalase (+/+) mice

Specific pathogen-free, catalase (−/−) and catalase (+/+) male mice, 8-10 weeks old, were used in this study as described previously [11, 29]. The housing, breeding, and experimental procedures were approved by the Animal Care Committees of Ewha Womans University (IACUC No.: ELAGC-07-1007) (Seoul, Korea).

### Pulmonary metastasis

Pulmonary metastasis was deemed to have occured in the animals following i.v. injection of B16F10 mouse melanoma cells derived from C57BL/6J mice, when visible, black, and round-shaped colonies appeared on lung surfaces. Two weeks after i.v. injection of B16F10 cells, their lungs were extirpated, and the number of black spherical B16F10 colonies was counted. In the first set of experiments, UCH-L1-overexpressing or control B16F10 cells were infected with Adv-vector or Adv-catalase. The infected cells were trypsinized, and suspended in PBS. Cells (1 × 10^6^) were injected intravenously into the tail veins of seven catalase (+/+) and seven catalase (−/−) mice. In the second set of experiments, UCH-L1-knocked down or control B16F10 cells (1.0 × 10^6^) were injected intravenously into the tail veins of male C57BL/6 catalase (+/+) and catalase (−/−) mice. Seven catalase (+/+) mice for control cells, five catalase (+/+) mice for UCH-L1-knocked down cells, seven catalase (−/−) mice for control cells, and seven catalase (−/−) mice for UCH-L1-knocked down cells were used.

### Statistical analysis

Data are expressed as mean ± standard deviation of the mean (SD) and analyzed using One-Way ANOVA on Originpro 7.5 software. A P value was derived to assess statistical significance.

## SUPPLEMENTARY MATERIAL FIGURES


