# Supplementary material for: Ubiquitin C-terminal hydrolase-L1 increases cancer cell invasion by modulating hydrogen peroxide generated via NADPH oxidase 4
Source: Oncotarget. 2015 Apr 15;6(18):16287–303. doi: 10.18632/oncotarget.3843 (PMC4599270; doi:10.18632/oncotarget.3843)
Supplement: Supplementary file 1 [file oncotarget-06-16287-s001.pdf]

Ubiquitin C-terminal hydrolase-L1 increases cancer cell invasion by modulating hydrogen peroxide generated via NADPH oxidase 4

Supplementary Material

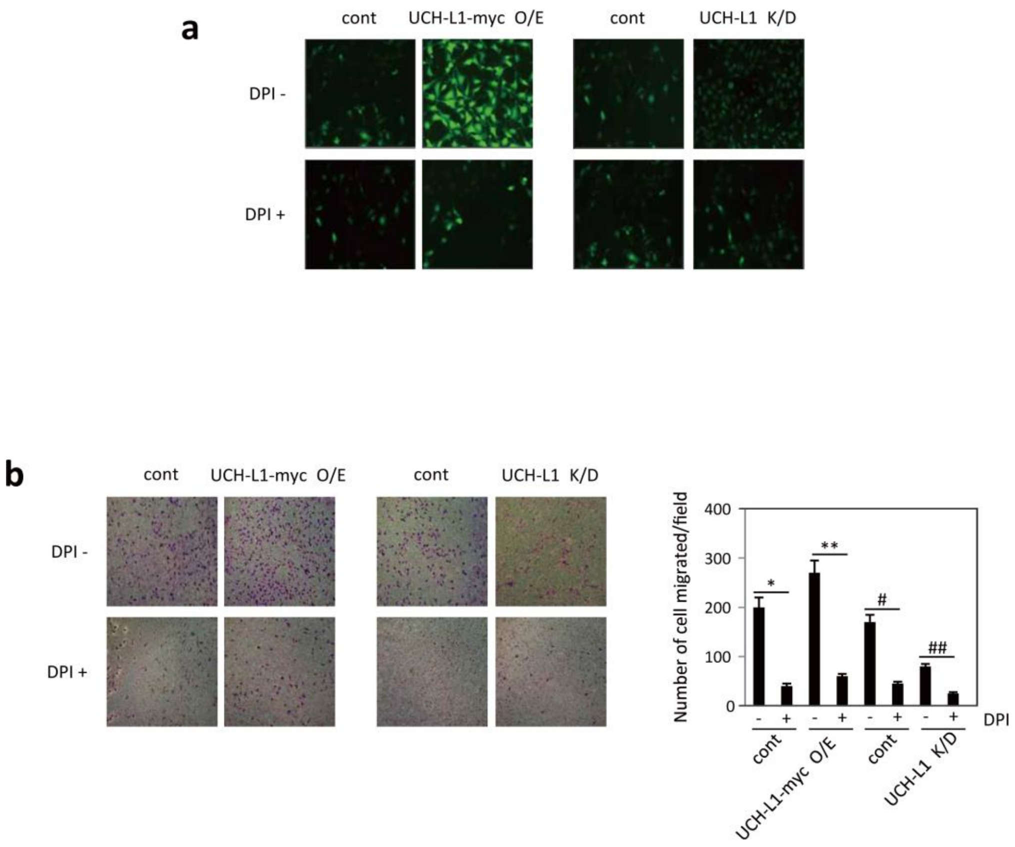

Supplementary Figure 1. The effects of UCH-L1 on cellular ROS-generation and cell invasion are attenuated by DPI.

(**a**) Cellular ROS level were measured after cells were treated with 20 mM of DPI for 30 min using 5 mM of CM-H_2DCFDA for 5 min. All experiments were performed in triplicates. (**b**) Cells treated with DPI (20 mM for 30 min) were seeded on matrigel-coated inserts in transwell chambers and incubated at 37°C for 24 h. Migrated cells were counted under a phase contrast microscope after staining with crystal violet. Bar graph shows the quantitative results. Data are mean ± SD (n = 3). *P<0.05 for cont (-DPI) vs. cont (+DPI), **P<0.05 for UCH-L1 O/E (-DPI) vs. UCH-L1 O/E (+DPI), $^{\#}$P<0.05 for cont (-DPI) vs. cont (+DPI), $^{\#\#}$P<0.05 for UCH-L1 K/D (-DPI) vs. UCH-L1 K/D (+DPI).

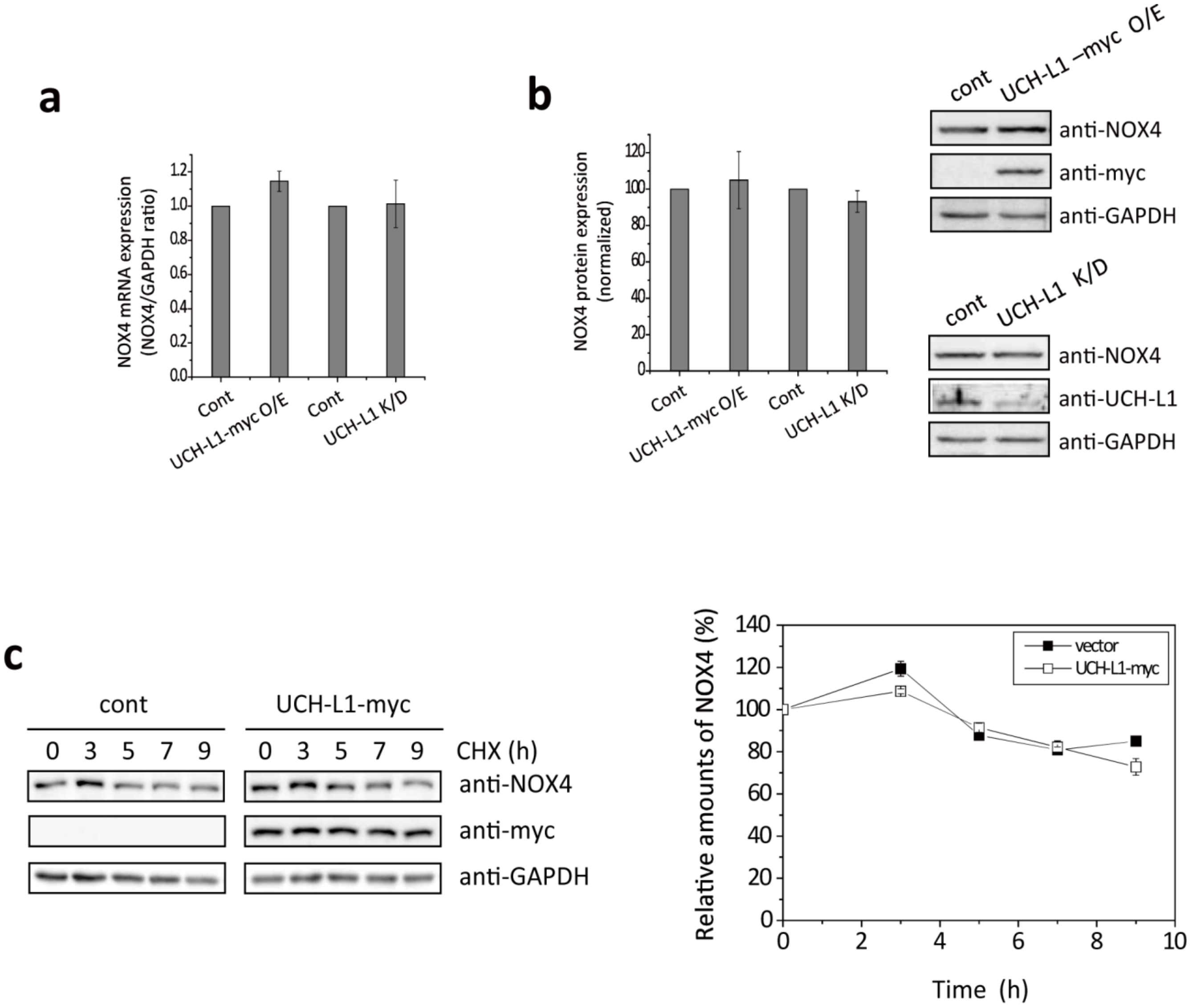

Supplementary Figure 2. UCH-L1 does not influence the expression and the half-life of NOX4. (**a**) Levels of NOX4 mRNA expression in UCH-L1-overexpressing or UCH-L1-knockdown B16F10 cells were measured by quantitative real-time RT-PCR. NOX4 mRNA was normalized to the house keeping gene, *GAPDH*. Data are mean ± SD (n = 3). (**b**) The protein expression of NOX4 in UCH-L1-overexpressing or UCH-L1-knockdown B16F10 cells was determined using western blotting with anti-NOX4 antibody. Data are mean ± SD (n = 3). (**c**) B16F10 cells were transfected with UCH-L1-myc expression vector or control vector and then cells were treated with 10 μg/mL cyclohexamide (CHX) for indicated time. The amounts of UCH-L1-myc, GAPDH, and endogenous NOX4 were measured by their specific antibodies and quantified by Multi-Gauge V3.0 software.

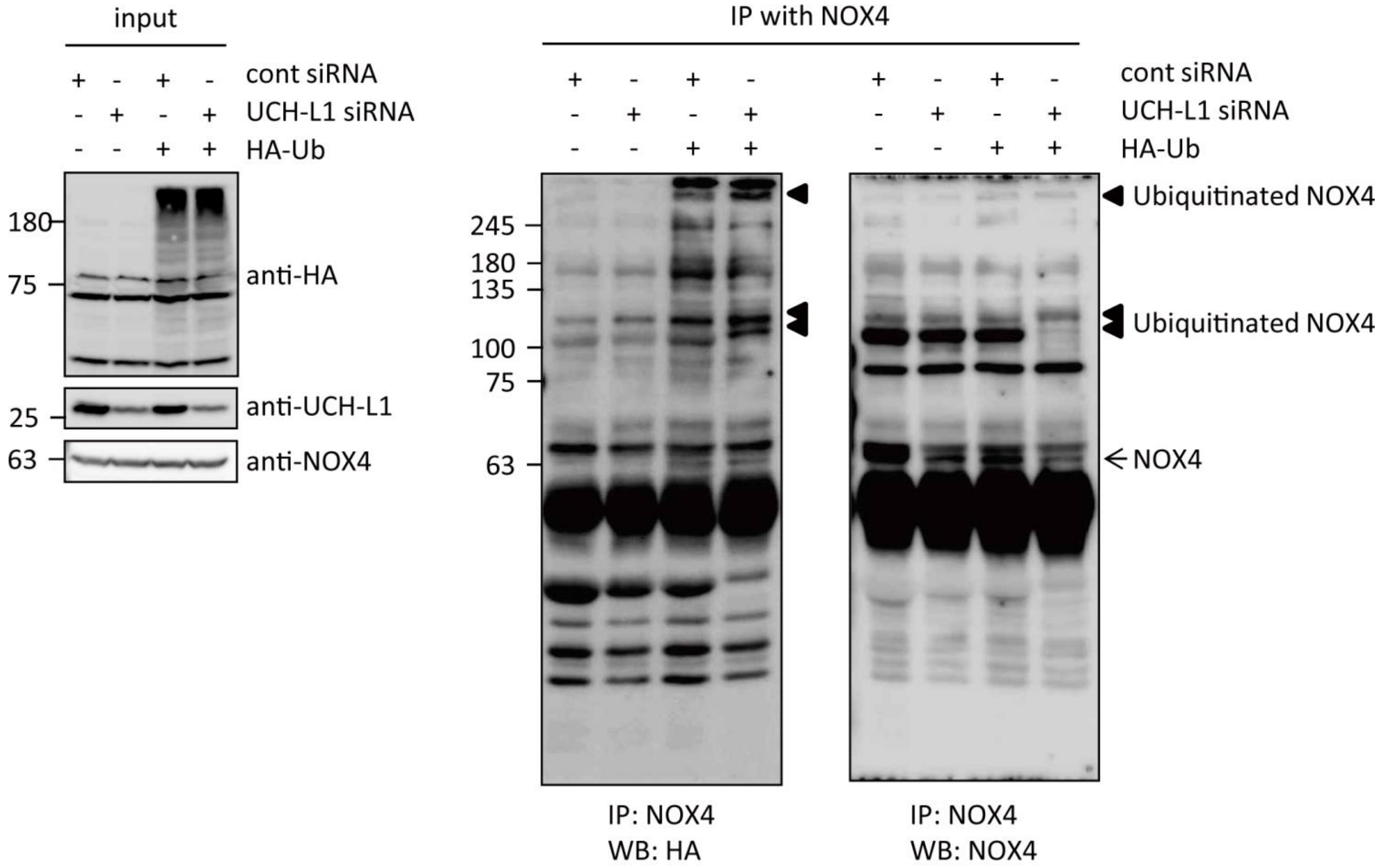

Supplementary Figure 3. UCH-L1 siRNA increases the level of ubiquitination of endogenous NOX4 in B16F10 cells.

B16F10 cells were firstly transfected with UCH-L1 siRNA or cont siRNA and after 48h, were secondly transfected with HA-Ub or control expression vectors. Levels of ubiquitination of endogenous NOX4 were determined by western blotting of immunoprecipitates with anti-NOX4 antibody (Proteintech Group, IL, USA). HA-Ub, UCH-L1, and NOX4 expressions were monitored by anti-HA, anti-UCH-L1, and anti-NOX4 antibodies, respectively. Arrow heads show ubiquitinated NOX4 and arrow indicates endogenous NOX4 in B16F10 cells.

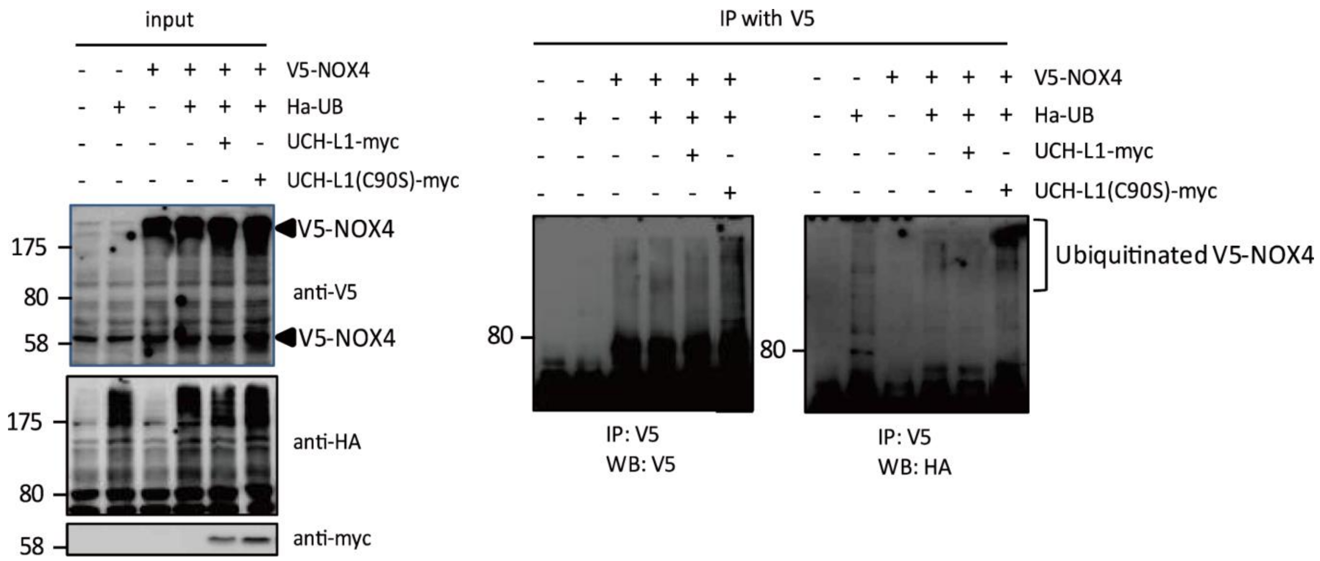

Supplementary Figure 4. UCH-L1(C90S), catalytically inactive mutant form of UCH-L1, has no effect on deubiquitination of NOX4 in HeLa cells.

HeLa cells were transfected with V5-NOX4, HA-Ub, UCH-L1-myc or UCH-L1(C90S)-myc expression vectors as indicated. Levels of ubiquitination of NOX4 were determined by western blotting of immunoprecipitates with anti-HA antibody, while NOX4 expression was monitored with anti-V5 antibody. Cells were lysed in IP buffer and then immunoprecipitated with anti-V5 antibody. Levels of ubiquitinated NOX4 and total protein levels of V5-NOX4, HA-Ub, and UCH-L1-myc were determined by western blotting. Arrow head show V5-NOX4.
